# Synthesis and *in Silico* Evaluation of Novel Compounds for PET-Based Investigations of the Norepinephrine Transporter

**DOI:** 10.3390/molecules20011712

**Published:** 2015-01-20

**Authors:** Catharina Neudorfer, Amir Seddik, Karem Shanab, Andreas Jurik, Christina Rami-Mark, Wolfgang Holzer, Gerhard Ecker, Markus Mitterhauser, Wolfgang Wadsak, Helmut Spreitzer

**Affiliations:** 1 Department of Biomedical Imaging and Image-guided Therapy, Division of Nuclear Medicine, Medical University of Vienna, Waehringer Guertel 18-20, 1090 Vienna, Austria; E-Mails: karem.shanab@gmail.com (K.S.); christina.rami-mark@meduniwien.ac.at (C.R.-M.); markus.mitterhauser@meduniwien.ac.at (M.M.); wolfgang.wadsak@meduniwien.ac.at (W.W.); 2 Division of Drug Synthesis, Department of Pharmaceutical Chemistry, Faculty of Life Sciences, University of Vienna, Althanstraße 14, 1090 Vienna, Austria; E-Mail: wolfgang.holzer@univie.ac.at; 3 Division of Drug Design and Medicinal Chemistry, Department of Pharmaceutical Chemistry, Faculty of Life Sciences, University of Vienna, Althanstraße 14, 1090 Vienna, Austria; E-Mails: amir.seddik@univie.ac.at (A.S.); andreas.jurik@univie.ac.at (A.J.); gerhard.f.ecker@univie.ac.at (G.E.)

**Keywords:** NET, ADHD, cocaine dependence, BAT, PET, FAPPI

## Abstract

Since the norepinephrine transporter (NET) is involved in a variety of diseases, the investigation of underlying dysregulation-mechanisms of the norepinephrine (NE) system is of major interest. Based on the previously described highly potent and selective NET ligand 1-(3-(methylamino)-1-phenylpropyl)-3-phenyl-1,3-dihydro-2*H*-benzimidaz- ol-2-one (Me@APPI), this paper aims at the development of several fluorinated methylamine-based analogs of this compound. The newly synthesized compounds were computationally evaluated for their interactions with the monoamine transporters and represent reference compounds for PET-based investigation of the NET.

## 1. Introduction

Abnormal regulation of the norepinephrine transporter (NET) or NET dysfunction, respectively, cause either increased or decreased levels of norepinephrine (NE) in the synaptic cleft. Since NE is a fundamental neurochemical messenger, its accurate regulation is of major importance. Thus, the NET, responsible for NE equilibrium in the synaptic cleft, is representing the reuptake site and considered to be involved in a variety of neurological/psychiatric disorders [1,2], but also plays a pivotal role in cardiovascular [1,2,3] and metabolic diseases [3,4,5]. Reduced NET levels go along with neurological disorders like major depression [6,7], Parkinson’s disease (PD), Alzheimer’s disease (AD) [8,9,10,11,12,13,14,15,16,17,18], and cardiovascular diseases such as hypertension, cardiomyopathy, and heart failure [5,13]. Furthermore, a dysfunction of the NE system was reported in Attention Deficit Hyperactivity Disorder (ADHD) [9,17,19], suicide [1,12,20], substance abuse (cocaine dependence) [16], and schizophrenia [10]. A more recent discovery is the involvement of the NET in diseases like diabetes and obesity, due to its presence in brown adipose tissue (BAT) and the proposed activation thereof via NE [4,5,21].

Based on the fact that the NET is involved in such a variety of diseases, the investigation of the underlying dysregulation-mechanism of the NE system is of major interest. For this purpose, information about the transporter abundance and density in healthy and pathological living human brains is required. The most suitable and accurate technique to gain this information is positron emission tomography (PET). As a non-invasive molecular imaging technique, it represents a suitable approach towards the collection of missing data in the living organism and direct quantification of receptor/transporter densities *in vivo*. To fully gain insight in the molecular changes of the noradrenergic system via PET, however, prior development of suitable NET PET radioligands is required.

So far, radiolabeled NET binding reboxetine analogs [^11^C]MeNER, [^11^C]MRB, ((*S,S*)-2-(α-(2-[^11^C]- methoxyphenoxy)benzyl)morpholine) and [^18^F]FMeNER-D_2_ ((*S,S*)-2-(α-(2-[^18^F]fluoro[^2^H_2_]methoxy- phenoxy)benzyl)morpholine) have been described, which however display certain limitations such as metabolic instability, complex radiosyntheses, or late equilibria [22,23,24,25,26].

**Figure 1:**
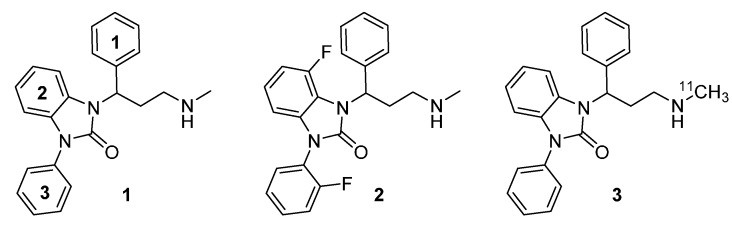
Structures of the highly potent and selective NET ligands 1-(3-(methylamino)- 1-phenylpropyl)-3-phenyl-1,3-dihydro-2*H*-benzimidazol-2-one (Me@APPI, **1**) and 4-fluoro- 1-(2-fluorophenyl)-3-(3-(methylamino)-1-phenylpropyl)-1,3-dihydro-2*H*-benzimidazol-2- one (**2**) as well as radiolabeled analog [^11^C]Me@APPI (**3**).

Recently, Zhang *et al.* [26] evaluated a series of benzimidazolone-based propanamines with *in vitro* inhibitory activity on the human norepinephrine transporter (hNET). The results of these investigations suggested that compounds containing a phenyl moiety directly attached at the benzimidazolone ring (e.g., **1**, Figure 1) were the most potent, representing a half maximal inhibitory concentration (IC_50_) below 10 nM (IC_50_ < 10 nM). Furthermore, hNET selectivity over human serotonin transporter (hSERT) turned out >300-fold superior to those of reboxetine and atomoxetine (16- and 81-fold) [26]. Fluorination at position 2 of the phenyl moiety attached to the benzimidazolone ring (e.g., **2**, Figure 1), indicated similar hNET potency, comparable to its non-fluorinated analogs (e.g., **1**) and additionally exhibited hNET selectivity over hSERT (80-fold) similar to atomoxetine [26].

Both benzimidazolone derived propanamines with a phenyl moiety (e.g., **1**), as well as a fluorinated phenyl moiety (as in **2**) indicated excellent hNET selectivity over human dopamine transporter (hDAT) with < 50% inhibition of the cocaine analog [^3^H]WIN-35,428, binding to hDAT at a concentration of 10 µM [26]. Given those findings, both described benzimidazolone-based propanamines **1** and **2** represent excellent candidates for selective and potent NET inhibition with high affinity and low unspecific binding. Thus, on the basis of the results of Zhang *et al.* [26] the methylamino moiety of the core compound **1** has been radiolabeled with ^11^C and tested by our research group [27]. All investigated preclinical parameters, such as affinity, blood brain barrier penetration, lipophilicity, metabolic degradation, and selectivity showed excellent results, thus suggesting suitability of ^11^C-radiolabeled 1-(3-(methylamino)-1-phenylpropyl)-3-phenyl-1,3-dihydro-2*H*-benzimidazole-2-one ([^11^C]Me@APPI) (**3**, Figure 1) as a NET radioligand for use in PET.

**Figure 2:**
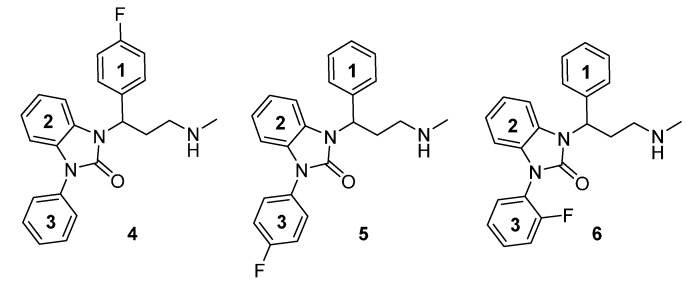
Chemical structure of envisaged reference compounds **4**–**6** (FAPPI:1-3).

Due to successful preclinical testing of [^11^C]Me@APPI (**3**) and given the excellent *in vitro* results of compound **2**, shown by Zhang *et al.* [26] the aim of this paper is the synthesis and docking studies of several fluorinated analogs **4**–**6** of compound **1** (Figure 2) as reference compounds for their later prepared radioactive analogs. All methylamine-derived benzimidazolone derivatives **4**–**6** will be subjected to affinity, selectivity, and lipophilicity studies towards the NET as well as blood brain barrier penetration experiments at the Medical University of Vienna. The most promising NET ligands will then be selected for the development of new, selective PET tracers for the NET and after radiolabeling with both ^11^C and ^18^F, they will be the subject of further experiments.

## 2. Results and Discussion

**Scheme 1:**
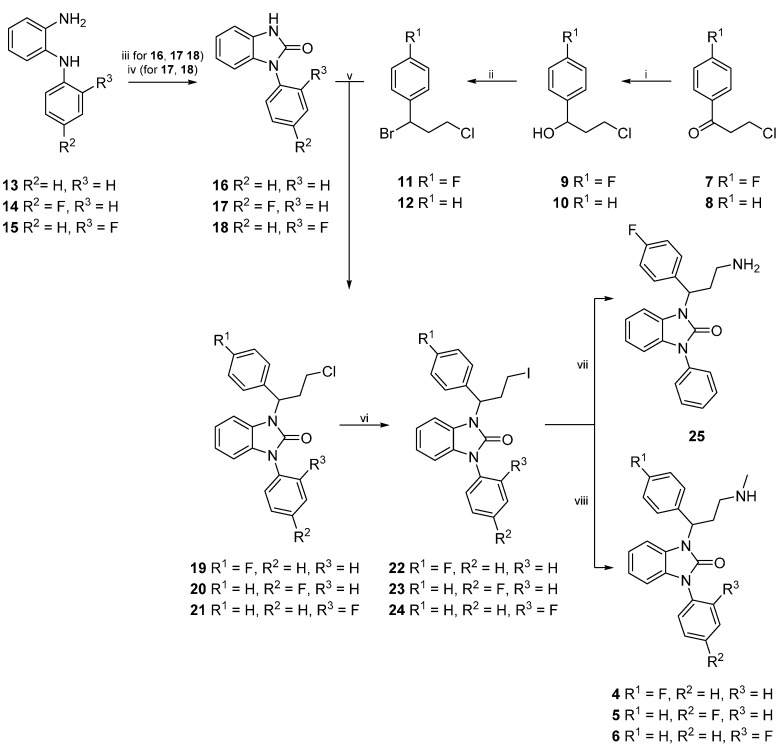
Synthesis of compounds **4**‒**6**.

The synthesis of reference compounds **4**–**6** first required the preparation of side chains **11** and **12**, as well as core compounds **16**–**18**. After successful preparation, side chain **11** was merged in a condensation reaction with core compound **16**, whereas side chain **12** was reacted with core compounds **17** and **18**, prior to halogen exchange and substitution with methylamine (Scheme 1).

*Reagents and conditions*: (i) THF, EtOH, NaBH_4_, −10 °C → −5 °C, 10 min, yields for **9**: 95%, **10**: 100%; (ii) aq. HBr, rt, 3 h, yields for **11**: 64%, **12**: 86%; (iii) 1,1'-carbonyldiimidazole, anhyd. THF, rt, overnight, yields for **16**: 74%, **17**: 61%, **18**: 69%; (iv) 1,1'-carbonyldiimidazole, anhyd. DMF, 90 °C, 2 h, yields for **17**: 80%, **18**: 80%; (v) K_2_CO_3_, DMF, rt, 30 min → addition of **11** and **12** → rt, 30 min, yields for **19**: 32%, **20**: 63%, **21**: 55%; (vi) NaI, acetone, reflux, 24 h, yields for **22**: 82%, **23**: 76%, **24**: 53%; (vii) NH_3_ in isopropanol, 80 °C, 3 h, yield for **25**: 50%; (viii) methylamine in EtOH, 80 °C, 3 h, yields for **4**: 48%, **5**: 29%, **6**: 30%.

For the synthesis of side chains **11** and **12**, a protocol of Varney *et al.* [28] was adopted and the keto group of commercially available compounds **7** and **8** was reduced with sodium borohydride in order to obtain intermediate alcohols **9** and **10**. Subsequent bromination of **9** and **10** with aqueous hydrogen bromide led to the formation of products **11** and **12**, respectively.

**Scheme 2:**
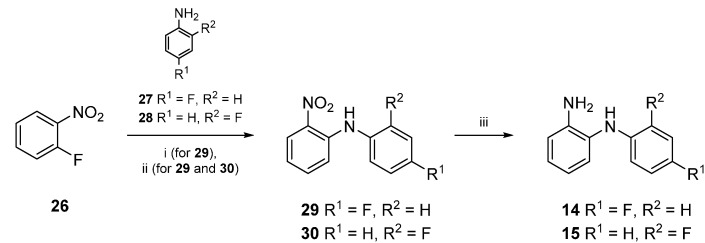
Synthesis of compounds **14**‒**15**.

Core compound **16** was prepared by the reaction of commercially available *N*-phenylbenzene-1,2-diamine (**13**) with 1,1'-carbonyldiimidazole. For the preparation of **17** and **18** however, **14** and **15** first had to be made accessible (Scheme 2). Thus, 1-fluoro-2-nitrobenzene (**26**) reacted with commercially available fluoroanilines **27** and **28**, respectively, to obtain disubstituted amines **29** and **30**. Therefore, two different methods were applied (Scheme 2): The first method (i) was conducted by heating **26** and **27** with anhydrous potassium fluoride and potassium carbonate in a microwave oven [29]. Since the adoption of an alternative method [30]—conventional heating at 180 °C—gave compound **29** in higher yields, this approach (ii) was chosen for the large scale synthesis of **29** as well as for the preparation of **30**.

*Reagents and conditions*: (i) anhyd. KF, K_2_CO_3_, microwave oven, 900 W, 10 min, yields for **29**: 58%; (ii) anhyd. KF, K_2_CO_3_, 180 °C, 2 d, yields for **29**: 68%, **30**: 52%; (iii) Zn, AcOH, 0 °C → rt, 2 h, yields for **14**: 93%, **15**: n.d.

In the next reaction step, the nitro groups of both disubstituted amines **29** and **30** were reduced. For this purpose **29** or **30** were added to a mixture of zinc/acetic acid. The resulting amines **14** or **15** were obtained in excellent yields (Scheme 2) [30].

Freshly prepared intermediates **14** and **15** were then subjected to a cyclization reaction with 1,1'-carbonyldiimidazole in DMF under anhydrous conditions (Scheme 1) by modifying a synthesis protocol according to Zhang *et al.* [26]. DMF was preferred over THF to ensure higher yields and shorter reaction times.

Condensation reactions of benzimidazolones **16**, **17**, and **18** with side chains **11** and **12**, respectively were performed under basic conditions (Scheme 1) by adapting a procedure of Jona *et al.* [31]. After purification, the chloro-substituted derivatives **19**–**21** were converted into the iodo compounds **22**–**24** in a Finkelstein reaction. Target compounds **4**–**6** (FAPPI:1-3) were then obtained by heating derivatives **22**–**24** in a solution of methylamine in ethanol in a sealed tube for 3 h. In addition to reference compounds **4**–**6**, free amine **25** was synthesized by dissolving **22** in a solution of ammonia in isopropanol and heating the resulting mixture in a sealed tube for 3 h. As compound **25** features a free amine moiety, it can be considered a precursor for radiosynthesis.

Since compounds **4** and **5** comprise a novel fluoro substitution, a computational docking study was performed to assess if these compounds still would fit in the binding site of the NET. Furthermore, we aimed at creating a binding mode hypothesis which allows gaining insights into the molecular basis of binding and selectivity towards the monoamine transporters. As the basic scaffold has been shown to act in an enantioselective manner, the respective (*R*) enantiomers were used throughout the docking studies [26]. The ligands were docked in the substrate binding site (S1) of the outward-open conformation of the transporter models (see Experimental Section for details), since related inhibitors, such as nortriptyline, sertraline, mazindol, *etc.* were also shown to fit in the S1 of the *Drosophila* DAT (dDAT) and the “SERT”-ized leucine transporter (“LeuBAT”) in the same protein conformation [32,33]. Interestingly, the co-crystallized ligand in dDAT, nortriptyline (**31**), has the same ranking of human NET, SERT and DAT activity as reference compound **1**, *i.e.*, 4.4, 18 and 1149 nM K_D_ *vs.* 9, 2995 and >10,000 nM IC_50_, respectively [26,34]. Additionally, nortriptyline shares important structural features with the benzimidazolones, *i.e.*, two aromatic moieties and an *N*-methyl-ethylenamine side chain. Therefore their binding mode can be expected to be similar.

Common scaffold clustering [35] revealed two binding hypotheses (see Experimental Section) which indicated that compounds **4**–**6** fit in the S1 of all three transporters. Hence, additional fluorination does not seem to cause steric clashes. In both hypotheses, the most prominent protein-ligand interaction was the cationic nitrogen atom placed in the A sub pocket [36], located between the central Asp75/98/79 side chain as a salt-bridge and the Phe72/95/76 side chain as a cation-*pi* interaction in NET/SERT/DAT, respectively. This is well in accordance with the X-ray structures of the templates. Additional *pi-pi* stacking interactions with Phe152/176/156 and Phe323/355/341 further promote the binding in both hypotheses obtained:

Hypothesis 1: the benzimidazolone heterocycle (ring 2) is placed in the B sub pocket and ring 3 in the C pocket, whereas ring 1 is solvent exposed (see figure in Experimental Section).

**Figure 3:**
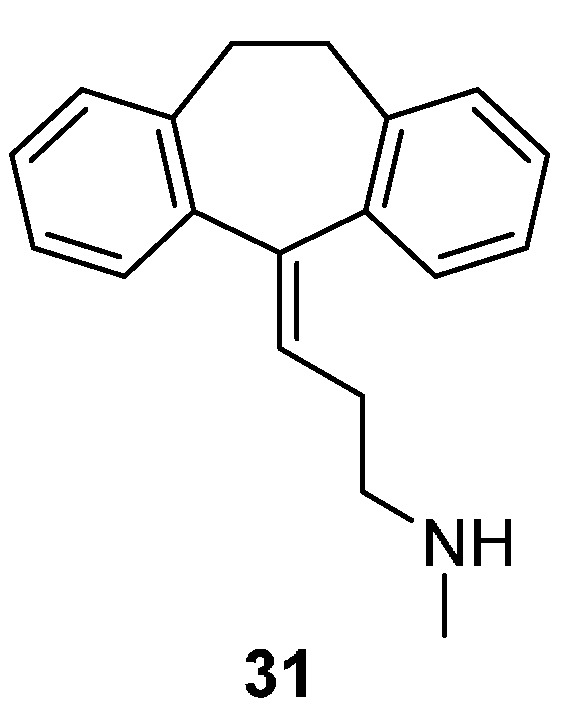
Chemical structure of nortriptyline (co-crystallized ligand from the template, PDB code 4M48) [28].

**Figure 4:**
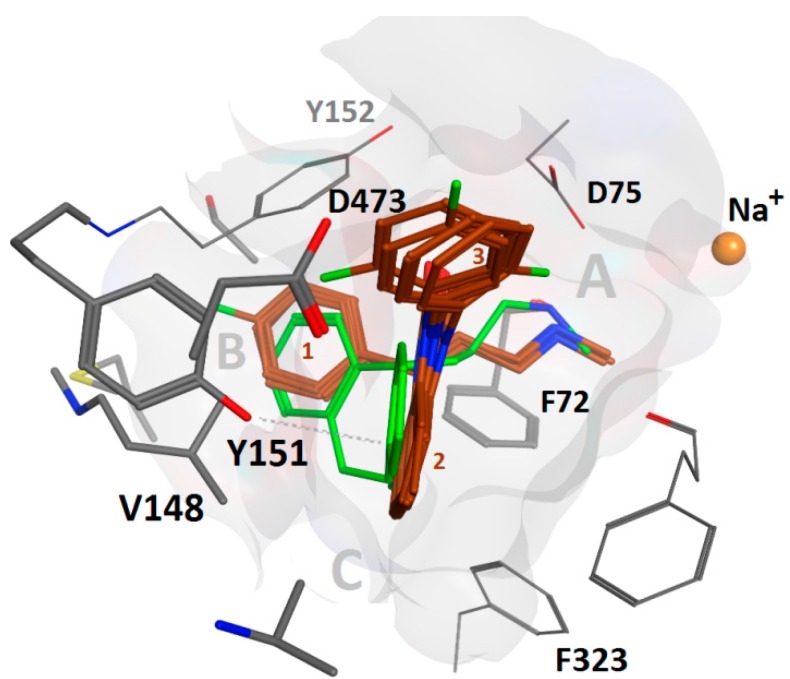
Overlay of compounds **1**, **2**, and **4**–**6** (maroon) in binding hypothesis 2 in hNET showing agreement with the co-crystal pose of nortriptyline (**31**) (green). Val148 and Asp473 allow more space than Ile172 and Glu439 in hSERT, resp., whereas Tyr151 might induce a more potent stacking interaction as compared to Phe155 in hDAT. The angle between ligand ring 2 and 3 is almost 90° in all poses. The extracellular space is located above.

Hypothesis 2: Ligand ring 1 is placed in the B pocket whereas ring 2 is placed at the same height (measured from the membrane-water interface) and overlap with the rings of nortriptyline (**31**, Figure 3). The solvent exposed Tyr151/Tyr175/Phe155 in NET/SERT/DAT, resp. T-stacks with ring 2 whereas ring 3 points extracellularly (Table 1, Figure 4).

**Table 1:** Sequence alignment of all distinct monoamine transporter residues located in sub sites (reference [32]) in the vicinity of the docked compounds. Red: hydrophilic side chain, green: lipophilic side chain, bold: bulkier side chain.

	B Site				C Site		Outer Site		
hNET	S420	M424	G149	V148	A145	F72	D473	Y151	A477
hSERT	T439	L443	A173	I172	A177	Y95	E493	Y175	T497
hDAT	A423	M427	G153	V152	S149	F76	D476	F155	A480

As binding hypothesis 2 is in close agreement with the co-crystallized ligands in dDAT and LeuBAT, we focus further analysis on this proposed binding mode. Binding hypothesis 2 indicates why the investigated compounds (**4**–**6**) show weaker affinity to SERT and DAT than to NET: lower SERT affinity may be due to Ile172 and Glu439, allowing less space for the ligand to be accommodated as compared to in NET, that comprises a valine and an aspartate at the homologous positions, respectively.

Lower DAT affinity could be ascribed to weaker T stacking interactions of Phe155 as compared to Tyr151 in NET, based on previous findings that a Tyr-Phe pair has a stronger binding energy than a Phe-Phe pair [37].

Since the docking studies indicate that fluorinated methyl amines **4**–**6** (FAPPI:1-3) bind in an analogous way to the NET as reference compound **1** [26], compounds **4**–**6** will be employed in future studies and evaluated for affinity and selectivity towards the NET. Additionally, lipophilicity studies and blood brain barrier penetration experiments are planned for compounds **4**–**6** at the Medical University of Vienna. The most promising derivatives regarding their suitability as NET ligands will then be selected for the further development of new and selective PET tracers for the NET, which will comprise either a [^11^C]methylamine, [^18^F]fluoroalkyl amine or [^18^F]fluorobenzene radiolabel, respectively. The results of ongoing studies on affinity, selectivity and lipophilicity of the discussed compounds, will be published in a subsequent paper.

## 3. Experimental Section

### 3.1. General

The NMR spectra were recorded from CDCl_3_ or DMSO-*d*_6_ solutions on a Bruker DPX200 spectrometer (200 MHz for ^1^H, 50 MHz for ^13^C) or on a Bruker Avance III 400 spectrometer (400 MHz for ^1^H, 100 MHz for ^13^C, 40 MHz for ^15^N, 376 MHz for ^19^F) at 25 °C. The center of the solvent (residual) signal was used as an internal standard which was related to TMS with δ 7.26 ppm (^1^H in CDCl_3_), δ 2.49 ppm (^1^H in DMSO-*d*_6_), δ 77.0 ppm (^13^C in CDCl_3_) and δ 39.5 ppm (^13^C in DMSO-*d*_6_). ^15^N NMR spectra (gs-HMBC, gs-HSQC) were referenced against neat, external nitromethane, ^19^F NMR spectra by absolute referencing via *Ξ* ratio. Digital resolutions were 0.25 Hz/data point in the ^1^H and 0.3 Hz/data point in the ^13^C-NMR spectra. Coupling constants (*J*) are quoted in Hz. The following abbreviations were used to show the multiplicities: s: singlet, d: doublet, t: triplet, q: quadruplet, dd: doublet of doublet, m: multiplet. Mass spectra were obtained on a Shimadzu QP 1000 instrument (EI, 70 eV), high-resolution mass spectrometry (HRMS) was carried out on a Finnigan MAT 8230 (EI, 10 eV) or Finnigan MAT 900 S (ESI, 4 kV, 3 μA, CH_3_CN/MeOH) electrospray ionization mass spectrometer with a micro-TOF analyzer. Microwave experiments were carried out in a Synthos 3000 microwave oven (SXQ80 rotor, Anton Paar, Graz, Austria) with an internal temperature probe. Compound purity: all compounds synthesized featured a purity of at least 95%.

### 3.2. Syntheses

#### 3.2.1. General Procedure for the Synthesis of **9** and **10**

Starting materials **7** or **8**, respectively (1 mmol) was dissolved in THF (1 mL) and EtOH (1 mL) was added. The mixture was cooled to −10 °C and NaBH_4_ (1.05 mmol) was slowly added at this temperature. The solution was stirred at −5 °C for 10 min and thereafter, poured into a mixture of saturated aqueous ammonium chloride (3 mL) in ice (1.5 g). The product was extracted with diethyl ether, dried over Na_2_SO_4_ and evaporated to dryness. The crude product was employed directly in the subsequent reaction step without further purification.

*3-Chloro-1-(4-fluorophenyl)propan-1-ol* (**9**). Yield: 4.78 g (95%), pale yellow oil, analytical data are in complete accordance with literature values [38].

*3-Chloro-1-phenylpropan-1-ol* (**10**). Yield: 4.61 g (99%), light yellow oil, analytical data are in complete accordance with literature values [28].

#### 3.2.2. General Procedure for the Synthesis of **11** and **12**

To starting material **9** or **10**, respectively (1 mmol) was added 48% aqueous HBr (3 mL) and the mixture was stirred for 3h at room temperature. Thereafter, the solution was poured into a mixture of K_2_CO_3_ (1 g) in ice (5.5 g) and additional solid K_2_CO_3_ was added for neutralization (pH 7). The crude reaction product was extracted with diethyl ether, the combined organic layers were dried with MgSO_4_ and evaporated to dryness. The crude product was employed directly in the subsequent reaction step without further purification.

*1-(1-Bromo-3-chloropropyl)-4-fluorobenzene* (**11**). Yield: 64%, pale yellow oil, analytical data are in complete accordance with literature values [39].

*(1-Bromo-3-chloropropyl)benzene* (**12**). Yield: 5.64 g (86%), yellow oil, analytical data are in complete accordance with literature values [28].

#### 3.2.3. General Procedure for the Synthesis of **29** and **30**

4-Fluoroaniline or 2-fluoroaniline, respectively (1 mmol), anhydrous KF (1 mmol), and K_2_CO_3_ (1 mmol) were well powdered with a mortar and a pestle, then 1-fluoro-2-nitrobenzene (1 mmol) was added and the mixture was stirred for 2 days at 180 °C. Thereafter, water (5 mL) and CH_2_Cl_2_ (5 mL) were added and the organic layer was washed with 10% HCl (5 mL) and brine (5 mL). The combined organic layers were dried over Na_2_SO_4_ and evaporated to dryness prior to purification by column chromatography.

*N-(4-Fluorophenyl)-2-nitroaniline* (**29**). Yield: 68%, dark orange crystals, mp. 82–83 °C, purification: silica gel 60, petroleum ether/ethyl acetate 9:1 and RP-18 silica gel, methanol/water 7:3, analytical data are in complete accordance with literature values [29].

*2-Fluoro-N-(2-nitrophenyl)aniline* (**30**). Yield: 52%, orange crystals, mp. 79–80 °C, purification: silica gel 60, petroleum ether/ethyl acetate 9:1, analytical data are in complete accordance with literature values [40].

#### 3.2.4. Alternative Procedure for the Synthesis of **29**

4-Fluoroaniline (7.89 g, 6.73 mL, 70.87 mmol), anhydrous KF (4.13 g, 70.87 mmol), and K_2_CO_3_ (9.81 g, 70.87 mmol) were well powdered with a mortar and a pestle, then 1-fluoro-2-nitrobenzene (10.00 g, 7.47 mL, 70.87 mmol) was added and the mixture was irradiated in the microwave (900 W, 10 min). Thereafter, water (8 mL) and CH_2_Cl_2_ (10 mL) were added and the organic layer was washed with 10% HCl (5 mL) and brine (5 mL). The combined organic layers were dried over Na_2_SO_4_ and evaporated to dryness prior to purification by column chromatography (silica gel 60, petroleum ether/ethyl acetate 9.5:0.5). Yield: 9.51 g (58%), dark orange crystals, mp. 82 °C–83 °C.

#### 3.2.5. General Procedure for the Synthesis of **14** and **15**

To a solution of Zn^0^ (13.8 mmol) in glacial acetic acid (1 mL) was added starting material **28** or **29** (1 mmol) at 0 °C under argon atmosphere. After the addition, the mixture was allowed to warm to room temperature and was stirred for 2 h. Zn^0^ was filtered off and the pH of the solution was adjusted to pH 9 with 2N NaOH. Thereafter, the aqueous layer was extracted three times with CH_2_Cl_2_, the combined organic layers were dried over MgSO_4_ and evaporated to dryness.

*N-(4-Fluorophenyl)benzene-1,2-diamine* (**14**). Yield: 93%, dark orange-reddish oil, purification: silica gel 60, petrol ether/ethyl acetate 9:1, analytical data are in complete accordance with literature values [30].

*N-(2-Fluorophenyl)benzene-1,2-diamine* (**15**). The crude reaction product was subjected to the next reaction step without further purification.

#### 3.2.6. General Procedure for the Synthesis of **16**–**18**

To a solution of starting materials **13**, **14**, or **15** (1 mmol) in THF was added 1,1'-carbonyldiimidazole (1.4 mmol) under argon atmosphere and the mixture was stirred at room temperature overnight. Thereafter, the crude reaction product was purified by column chromatography.

*1-Phenyl-1,3-dihydro-2H-benzimidazol-2-one* (**16**). Yield: 0.85 g (74%), pink crystals, mp. 201 °C–202 °C, THF: 10 mL, purification: silica gel 60, petroleum ether/ethyl acetate 1:1, analytical data are in complete accordance with literature values [41].

*1-(4-Fluorophenyl)-1,3-dihydro-2H-benzimidazol-2-one* (**17**). Yield: 61%, brown resin, THF: 25 mL, purification: silica gel 60, petroleum ether/ethyl acetate 9:1, ^1^H-NMR (200 MHz, CDCl_3_): δ (ppm) 6.74–6.81 (m, 2H), 6.89–7.11 (m, 2H), 7.14–7.23 (m, 2H), 7.48–7.59 (m, 1H), 7.73–7.78 (m, 1H), 9.03 (br s, 1H), ^13^C-NMR (50 MHz, CDCl_3_): δ (ppm) 108.5, 110.0, 116.3, 116.8, 121.4, 121.7, 122.3, 128.0, 128.2, 130.4, 135.1, 155.1, 159.3, 164.2, MS: *m/z* (%) 228 (M^+^, 100), 199 (31), 172 (8), 114 (9), 95 (10), 75 (17), 51 (10), HRMS: *m/z* calculated for C_13_H_10_FN_2_O [M + H]^+^: 229.0772. Found: 229.0769.

*1-(2-Fluorophenyl)-1,3-dihydro-2H-benzimidazol-2-one* (**18**). Yield: 69%, brown resin, THF: 20 mL, purification: silica gel 60, petroleum ether/ethyl acetate 9:1, ^1^H-NMR (200 MHz, CDCl_3_): δ (ppm) 6.82–6.85 (m, 1H), 7.00–7.19 (m, 3H), 7.26–7.88 (m, 2H), 7.43–7.60 (m, 2H), 10.54 (br s, 1H), ^13^C-NMR (50 MHz, CDCl_3_): δ (ppm) 108.9, 110.1, 117.0, 117.4, 121.5, 121.9, 122.4, 124.9, 125.0, 128.2, 129.6, 130.4, 154.8, 155.4, 160.5, MS: *m/z* (%) 228 (M^+^, 33), 199 (9), 181 (15), 149 (17), 111 (22), 97 (20), 71 (41), 69 (100), 55 (53), 43 (56), HRMS: *m/z* calculated for C_13_H_9_FN_2_NaO [M + Na]^+^: 251.0597. Found: 251.0592.

#### 3.2.7. Alternative Procedure for the Synthesis of **17** and **18**

A solution of 1,1'-carbonyldiimidazole (1.4 mmol) in DMF (4 mL) was slowly added to a mixture of **14** or **15** (1 mmol) in DMF (4 mL) under argon atmosphere. The resulting solution was stirred at 90 °C for 2 h. After completion of the reaction, the solvent was evaporated *in vacuo*, the slurry was taken up in water, filtered and dried.

*1-(4-Fluorophenyl)-1,3-dihydro-2H-benzimidazol-2-one* (**17**). Yield: 80%, brown resin.

*1-(2-Fluorophenyl)-1,3-dihydro-2H-benzimidazol-2-one* (**18**). Yield: 80%, brown resin.

#### 3.2.8. General Procedure for the Synthesis of **19–21**

Starting materials **16**–**18** (1 mmol) and K_2_CO_3_ (2 mmol) were suspended in DMF (1.8 mL) and stirred at 25 °C for 30 min. **11** and **12**, respectively (1.5 mmol) were added after 30 min and the solution was stirred at room temperature overnight. To the mixture was added ethyl acetate (5 mL) and water (5 mL). The aqueous layer was extracted several times with ethyl acetate (10 mL) and the combined organic layers were washed with brine, dried over MgSO_4_ and evaporated to dryness.

*1-(3-Chloro-1-(4-fluorophenyl)propyl)-3-phenyl-1,3-dihydro-2H-benzimidazol-2-one* (**19**). Yield: 32%, white oil, purification: silica gel 60, petroleum ether/ethyl acetate 8:2, ^1^H-NMR (200 MHz, CDCl_3_): δ (ppm) 2.70–2.87 (m, 1H), 3.12–3.30 (m, 1H), 3.60–3.66 (m, 2H), 5.73 (m, 1H), 7.00–7.11 (m, 6H), 7.38–7.57 (m, 7H), ^13^C-NMR (50 MHz, CDCl_3_): δ (ppm) 34.3, 42.0, 53.8, 108.7, 109.0, 115.5, 115.9, 121.7, 122.0, 126.0, 127.8, 129.2, 129.4, 129.5, MS: *m/z* (%) 380 (M^+^, 21), 210 (100) (M^+^-C_9_H_9_ClF), 181 (8), 167 (12), 135 (9), 115 (5), 109 (58), 77 (12), HRMS: *m/z* calculated for C_22_H_18_ClFN_2_ONa [M + Na]^+^: 403.0989. Found: 403.0989.

*1-(3-Chloro-1-phenylpropyl)-3-(4-fluorophenyl)-1,3-dihydro-2H-benzimidazol-2-one* (**20**). Yield: 63%, dark orange resin, purification: silica gel 60, petroleum ether/ethyl acetate 9:1 and RP-18 silica gel, methanol, ^1^H-NMR (400 MHz, CDCl_3_): δ (ppm) 2.80–2.88 (m, 1H, 2'-CH_2_), 3.17–3.26 (m, 1H, 2'-CH_2_), 3.62–3.70 (m, 2H, 3'-CH_2_), 5.79 (dd, *J* = 10.0 Hz and 5.6 Hz, 1H, 1'-CH), 7.04–7.09 (m, 4H, benzim 4-CH, benzim 5-CH, benzim 6-CH, benzim 7-CH), 7.21–7.26 (m, 2H, f-phen 3-CH, f-phen 5-CH), 7.31–7.33 (m, 1H, phen 4-CH), 7.36–7.40 (m, 2H, phen 3-CH, phen 5-CH), 7.52–7.56 (m, 4H, f-phen 2-CH, f-phen 6-CH, phen 2-CH, phen 6-CH), ^13^C-NMR (100 MHz, CDCl_3_): δ (ppm) 34.1 (2'-CH_2_), 42.0 (3'-CH_2_), 54.4 (1'-CH), 108.6 (benzim 4-CH), 109.0 (benzim 7-CH), 116.4 (d, *J* = 22.9 Hz, f-phen 3-CH, 116.4 (d, *J* = 22.9 Hz, f-phen 5-CH), 121.6 (benzim 5-CH), 122.1 (benzim 6-CH), 127.4 (phen 2-CH), 127.4 (phen 6-CH), 127.9 (d, *J* = 8.6 Hz, f-phen 2-CH), 127.9 (d, *J* = 8.6 Hz, f-phen 6-CH), 128.1 (phen 4-CH), 128.7 (benzim 7a-C), 128.8 (phen 3-CH), 128.8 (phen 5-CH), 129.4 (benzim 3a-C), 130.3 (d, *J* = 3.1 Hz, f-phen 1-C), 138.4 (phen 1-C), 153.2 (benzim 2-CO), 161.6 (d, *J* = 247.7 Hz, f-phen 4-CF), ^19^F-NMR (471 MHz, CDCl_3_): δ (ppm) -113.31 (m, 5-CF), MS: *m/z* (%) 380 (M^+^, 2), 228 (100) (M^+^-C_9_H_10_Cl), 199 (11), 185 (16), 153 (6), 117 (14), 91 (73), 75 (8), HRMS: *m/z* calculated for C_22_H_19_ClFN_2_O [M + H]^+^: 381.1170. Found: 381.1176.

*1-(3-Chloro-1-phenylpropyl)-3-(2-fluorophenyl-1,3-dihydro-2H-benzimidazol-2-one* (**21**). Yield: 55%, orange resin, purification: silica gel 60, petroleum ether/ethyl acetate 9:1, ^1^H-NMR (400 MHz, CDCl_3_): δ (ppm) 2.78–2.86 (m, 1H, 2'-CH_2_), 3.23 (br s, 1H, 2'-CH_2_), 3.64–3.68 (m, 2H, 3'-CH_2_), 5.79 (br s, 1H, 1'-CH), 6.85–6.87 (m, 1H, benzim 4-CH) 7.05–7.06 (m, 3H, benzim 5-CH, benzim 6-CH, benzim 7-CH), 7.28–7.40 (m, 5H, f-phen 3-CH, f-phen 6-CH, phen 3-CH, phen 4-CH, phen 5-CH), 7.43–7.48 (m, 1H, f-phen 4-CH), 7.52–7.56 (m, 3H, f-phen 5-CH, phen 2-CH, phen 6-CH), ^13^C-NMR (100 MHz, CDCl_3_): δ (ppm) 34.2 (2'-CH_2_), 41.9 (3'-CH_2_), 54.5 (1'-CH), 108.8 (d, *J* = 1.7 Hz, benzim 4-CH), 109.0 (benzim 7-CH), 117.1 (d, *J* = 19.5 Hz, f-phen 3-CH), 121.6 (benzim 5-CH), 121.9 (f-phen 1-C), 122.0 (benzim 6-CH), 124.8 (d, *J* = 3.9 Hz, f-phen 6-CH), 127.3 (phen 2-CH), 127.3 (phen 6-CH), 128.1 (phen 4-CH), 128.8 (phen 3-CH), 128.8 (phen 5-CH), 129.4 (benzim 3a-C), 129.5 (f-phen 5-CH), 130.2 (d, *J* = 7.8 Hz, f-phen 4-CH), 138.4 (phen 1-C), 153.0 (benzim 2-CO), 157.9 (d, *J* = 253.2 Hz, f-phen 2-CF), due to limited resolution of the measuring apparatus, quaternary carbon benzim 7a-C could not be detected, ^19^F-NMR (471 MHz, CDCl_3_): δ (ppm) -118.39 (m, f-phen 2-CF), MS: *m/z* (%) 380 (M^+^, 12), 228 (100) (M^+^-C_9_H_10_Cl), 199 (5), 153 (3), 117 (7), 91 (49), 75 (5), HRMS: *m/z* calculated for C_22_H_19_ClFN_2_O [M + H]^+^: 381.1170. Found: 381.1164.

#### 3.2.9. General Procedure for the Synthesis of **22–24**

A solution of starting materials **19**, **20**, or **21** (1 mmol) and NaI (1.03 g, 6.89 mmol) in acetone (7 mL) was refluxed for 24 h. The precipitate formed was filtered and the solvent was removed *in vacuo*.

*1-(1-(4-Fluorophenyl)-3-iodopropyl)-3-phenyl-1,3-dihydro-2H-benzimidazol-2-one* (**22**). Yield: 82%, yellow resin, ^1^H-NMR (200 MHz, CDCl_3_): δ (ppm) 2.77–2.92 (m, 1H), 3.14–3.31 (m, 3H), 5.63–5.70 (m, 1H), 7.01–7.10 (m, 6H), 7.36–7.56 (m, 7H), ^13^C-NMR (50 MHz, CDCl_3_): δ (ppm) 2.3, 35.3, 57.0, 108.8, 108.9, 115.5, 115.9, 121.6, 122.0, 126.0, 127.7, 128.5, 129.1, 129.3, 129.4, 134.0, 134.1, 134.3, 153.0, 159.9, 164.8, MS: *m/z* (%) 472 (M^+^, 32), 317 (8), 210 (100) (M^+^-C_9_H_9_F), 181 (11), 167 (23), 140 (3), 135 (43), 115 (8), 109 (34), 77 (15), 51 (7), HRMS: *m/z* calculated for C_22_H_18_FIN_2_ONa [M + Na]^+^: 495.0346. Found: 495.0353.

*1-(4-Fluorophenyl)-3-(3-iodo-1-phenylpropyl)-1,3-dihydro-2H-benzimidazol-2-one* (**23**). Yield: 76%, yellow crystals, mp. 39 °C–41 °C, purification: silica gel 60, petrol ether/ethyl acetate 9:1, ^1^H-NMR (200 MHz, CDCl_3_): δ (ppm) 2.81–2.99 (m, 1H, 2'-CH_2_), 3.11–3.35 (m, 3H, 2'-CH_2_, 3'-CH_2_), 5.70 (dd, *J* = 6 Hz and 2 Hz, 1H, 1'-CH), 7.04–7.09 (m, 4H, benzim 4-CH, benzim 5-CH, benzim 6-CH, benzim 7-CH), 7.17–7.42 (m, 5H, f-phen 3-CH, f-phen 5-CH, phen 4-CH, phen 2-CH, phen 5-CH), 7.51–7.57 (m, 4H, f-phen 2-CH, f-phen 6-CH, phen 2-CH, phen 6-CH), ^13^C-NMR (50 MHz, CDCl_3_): δ (ppm) 2.4 (2'-CH_2_), 35.2 (3'-CH_2_), 57.6 (1'-CH), 108.6 (benzim 4-CH), 109.2 (benzim 7-CH), 116.4 (d, *J* = 23 Hz, f-phen 3-CH), 116.4 (d, *J* = 23 Hz, f-phen 5-CH), 121.6 (benzim 5-CH), 122.1 (benzim 6-CH), 127.3 (phen 2-CH), 127.3 (phen 6-CH), 127.8 (phen 4-CH), 128.1 (d, *J* = 5 Hz, f-phen 2-CH), 128.1 (d, *J* = 5 Hz, f-phen 6-CH), 128.5 (benzim 7a-C), 128.8 (phen 3-CH), 128.8 (phen 5-CH), 129.4 (benzim 3a-C), 130.3 (d, *J* = 3Hz, f-phen 1-C), 138.1 (phen 1-C), 153.2 (benzim 2-CO), 161.6 (d, *J* = 247 Hz, f-phen 4-CF), MS: *m/z* (%) 472 (M^+^, 13), 317 (5), 228 (100) (M^+^-C_9_H_10_I), 199 (8), 185 (15), 117 (47), 103 (2), 91 (40), 75 (8), 55 (5), HRMS: *m/z* calculated for C_22_H_19_FIN_2_O [M + H]^+^: 473.0526. Found: 473.0506.

*1-(2-Fluorophenyl)-3-(3-iodo-1-phenylpropyl)-1,3-dihydro-2H-benzimidazol-2-one* (**24**). Yield: 53%, yellow crystals, mp. 38 °C–39 °C, purification: silica gel 60, petrol ether/ethyl acetate 9:1, ^1^H-NMR (200 MHz, CDCl_3_): δ (ppm) 2.80–2.98 (m, 1H, 2'-CH_2_), 3.13–3.36 (m, 3H, 2'-CH_2_, 3'-CH_2_), 5.67–5.74 (m, 1H, 1'-CH), 6.85–6.88 (m, 1H, benzim 4-CH) 6.99–7.07 (m, 3H, benzim 5-CH, benzim 6-CH, benzim 7-CH), 7.26–7.45 (m, 5H, f-phen 3-CH, f-phen 6-CH, phen 3-CH, phen 4-CH, phen 5-CH), 7.47–7.58 (m, 4 H, f-phen 4-CH, f-phen 5-CH, phen 2-CH, phen 6-CH), ^13^C-NMR (50 MHz, CDCl_3_): δ (ppm) 2.3 (2'-CH_2_), 35.4 (3'-CH_2_), 57.6 (1'-CH), 108.9 (d, *J* = 1.5 Hz, benzim 4-CH), 109.1 (benzim 7-CH), 117.1 (d, *J* = 19 Hz, f-phen 3-CH), 121.6 (benzim 5-CH), 121.9 (f-phen 1-C), 122.0 (benzim 6-CH), 124.8 (d, *J* = 3.5 Hz, f-phen 6-CH), 127.3 (phen 2-CH), 127.3 (phen 6-CH), 128.1 (phen 4-CH), 128.8 (phen 3-CH), 128.8 (phen 5-CH), 129.5 (f-phen 5-CH), 130.2 (d, *J* = 8 Hz, f-phen 4-CH), 138.2 (phen 1-C), 153.0 (benzim 2-CO), 157.8 (d, *J* = 251.5 Hz, f-phen 2-CF), MS: *m/z* (%) 472 (M^+^, 9), 317 (5), 241 (4), 228 (100) (M^+^-C_9_H_10_I), 199 (5), 185 (10), 117 (37), 91 (29), 75 (7), HRMS: *m/z* calculated for C_22_H_19_FIN_2_O [M + H]^+^: 473.0526. Found: 473.0532.

#### 3.2.10. General procedure for the synthesis of *1-(3-amino-1-(4-fluorophenyl)propyl)-3-phenyl-1,3- dihydro-2H-benzimidazol-2-one* (**25**)

1-(1-(4-fluorophenyl)-3-iodopropyl)-3-phenyl-1,3-dihydro-2*H*-benzimidazol-2-one (0.26 g, 0.55 mmol) and a solution of NH_3_ in isopropanol (2 M, 22 mL) were heated in a sealed tube for 3 h at 80 °C. After evaporation of the solvent, the crude product was purified by column chromatography (silica gel 60, CH_2_Cl_2_/MeOH 9:1). Yield: 0.10 g (50%), light brown crystals, mp. 87–88 °C. ^1^H-NMR (400 MHz, CDCl_3_): δ (ppm) 2.74–2.84 (m, 3H, 2'-CH_2_, 3'-CH_2_), 2.98–3.04 (m, 1H, 3'-CH_2_), 5.74–5.78 (m, 1H, 1'-CH), 6.79–6.81 (m, 1H, benzim 7-CH), 6.96–7.01 (m, 5H, benzim 4-CH, benzim 5-CH, benzim 6-CH, f-phen 3-CH, f-phen 5-CH), 7.30–7.33 (m, 1H, phen 4-CH), 7.40–7.48 (m, 4H, f-phen 2-CH, f-phen 6-CH, phen 3-CH, phen 5-CH), 7.52–7.54 (m, 2H, phen 2-CH, phen 6-CH), due to limited resolution of the instrumentation, the NH_2_ protons could not be detected, ^13^C-NMR (100 MHz, CDCl_3_): δ (ppm) 30.3 (2'-CH_2_), 37.8 (3'-CH_2_), 52.7 (1'-CH), 109.2 (benzim 4-CH), 110.0 (benzim 7-CH), 115.7 (d, *J* = 21.5 Hz, f-phen 3-CH), 115.7 (d, *J* = 8.2 Hz, f-phen 5-CH), 122.0 (benzim 5-CH), 122.3 (benzim 6-CH), 126.4 (phen 2-CH), 126.4 (phen 6-CH), 127.5 (benzim 7a-C), 128.1 (phen 4-CH), 129.2 (d, *J* = 8.2 Hz, f-phen 2-CH), 129.2 (d, *J* = 8.2 Hz, f-phen 6-CH), 129.5 (benzim 3a-C), 129.7 (phen 3-CH), 129.7 (phen 5-CH), 133.3 (d, *J* = 3.2 Hz, f-phen 1-C), 134.0 (phen 1-CH), 153.8 (benzim 2-CO), 162.3 (d, *J* = 247.4 Hz, f-phen 4-CF), ^19^F-NMR (471 MHz, CDCl_3_): δ (ppm) -113.68 (m, f-phen CF), MS: *m/z* (%) 361 (M^+^, 17), 331 (10), 210 (100) (M^+^-C_9_H_11_FN), 181 (15), 167 (16), 149 (29), 128 (17), 77 (19), 57 (20), 43 (12), HRMS: *m/z* calculated for C_22_H_21_FN_3_O [M + H]^+^: 362.1669. Found: 362.1674.

#### 3.2.11. General Procedure for the Synthesis of **4–6**

Starting materials **18**, **19** or **20** (1 mmol) and a solution of methylamine in EtOH (12.5 mL, 8 M) were heated in a sealed tube for 3 h at 80 °C. After evaporation of the solvent, the crude reaction product was purified by column chromatography.

*1-(1-(4-Fluorophenyl)-3-(methylamino)propyl)-3-phenyl-1,3-dihydro-2H-benzimidazol-2-one* (**4**). Yield: 48%, light orange resin, purification: silica gel 60, dichloromethane/methanol 9:1 and dichloromethane/ethyl acetate/methanol 7:2:1, ^1^H-NMR (400 MHz, CDCl_3_): δ (ppm) 2.42 (s, 3H, NHCH_3_), 2.57–2.74 (m, 4H, 2'-CH_2_, 3'-CH_2_), 3.15 (br s, 1H, NHCH_3_), 5.76–5.79 (m, 1H, 1'-CH), 6.88–6.90 (m, 1H, benzim 7-CH), 6.97–7.05 (m, 4H, benzim 5-CH, benzim 6-CH, f-phen 3-CH, f-phen 5-CH), 7.07–7.10 (m, 1H, benzim 4-CH), 7.39–7.43 (m, 1H, phen 4-CH), 7.46–7.57 (m, 6H, f-phen 2-CH, f-phen 6-CH, phen 2-CH, phen 3-CH, phen 5-CH, phen 6-CH), ^13^C-NMR (100 MHz, CDCl_3_): δ (ppm) 30.6 (2'-CH_2_), 35.9 (NHCH_3_), 48.3 (3'-CH_2_), 53.1 (1'-CH), 108.9 (benzim 4-CH), 109.5 (benzim 7-CH), 115.5 (d, *J* = 21.5 Hz, f-phen 3-CH), 115.5 (d, *J* = 21.5 Hz, f-phen 5-CH) 121.4 (benzim 5-CH), 121.8 (benzim 6-CH), 126.0 (phen 2-CH), 126.0 (phen 6-CH), 127.7 (phen 4-CH), 128.0 (benzim 7a-C), 129.0 (d, *J* = 8.1 Hz, f-phen 2-CH), 129.0 (d, *J* = 8.1 Hz, f-phen 6-CH), 129.4 (benzim 3a-C), 129.5 (phen 3-CH), 129.5 (phen 5-CH), 134.4 (phen 1-CH), 134.6 (d, *J* = 3.4 Hz, f-phen 1-CH), 153.5 (benzim 2-CO), 162.1 (d, *J* = 246.7 Hz, f-phen 4-CF), ^19^F-NMR (471 MHz, CDCl_3_): δ (ppm) -114.36 (m, f-phen 4-CF), MS: *m/z* (%) 375 (M^+^, 16), 210 (57) (M^+^-C_10_H_13_FN), 181 (10), 167 (12), 150 (10), 109 (22), 97 (16), 71 (27), 57 (78), 44 (100), HRMS: *m/z* calculated for C_23_H_23_FN_3_O [M + H]^+^: 376.1825. Found: 376.1821.

*1-(4-Fluorophenyl)-3-(3-(methylamino)-1-phenylpropyl)-1,3-dihydro-2H-benzimidazol-2-one* (**5**). Yield: 29%, light yellow crystals, mp. 100 °C–102 °C, purification: silica gel 60, dichloromethane/methanol 9:1 and RP-18 silica gel methanol/water 9:1 and 7:3, ^1^H-NMR (200 MHz, CDCl_3_): δ (ppm) 2.53 (s, 3H, NHCH_3_), 3.13 (br s, 4H, 2'-CH_2_, 3'-CH_2_), 5.77–5.81 (m, 1H, 1'-CH), 6.96–7.03 (m, 4H, benzim 4-CH, benzim 5-CH, benzim 6-CH, benzim 7-CH), 7.16–7.34 (5H, f-phen 3-CH, f-phen 5-CH, phen 4-CH, phen 2-CH, phen 5-CH), 7.49–7.56 (m, 4H, f-phen 2-CH, f-phen 6-CH, phen 2-CH, phen 6-CH), due to limited resolution of the instrumentation, the NH proton could not be detected, ^13^C-NMR (50 MHz, CDCl_3_): δ (ppm) 27.6 (2'-CH_2_), 33.1 (NHCH_3_), 47.0 (3'-CH_2_), 54.2 (1'-CH), 108.8 (benzim 4-CH), 109.9 (benzim 7-CH), 116.6 (d, *J* = 23 Hz, f-phen 3-CH), 116.6 (d, *J* = 23 Hz, f-phen 5-CH), 122.1 (benzim 5-CH), 122.6 (benzim 6-CH), 127.2 (phen 2-CH), 127.2 (phen 6-CH), 127.7 (phen 4-CH), 128.3 (d, *J* = 6 Hz, f-phen 2-CH), 128.3 (d, *J* = 6 Hz, f-phen 6-CH), 128.4 (benzim 7a-C), 128.9 (phen 3-CH), 128.9 (phen 5-CH), 129.2 (benzim 3a-C), 129.7 (d, *J* = 3 Hz, f-phen 1-C), 136.9 (phen 1-C), 153.4 (benzim 2-CO), 161.7 (d, *J* = 248 Hz, f-phen 4-CF), MS: *m/z* (%) 375 (M^+^, 11), 330 (7), 228 (50) (M^+^-C_10_H_14_N), 199 (7), 185 (9), 147 (17), 128 (26), 117 (8), 91 (13), 58 (34), 44 (100), HRMS: *m/z* calculated for C_23_H_23_FN_3_O [M + H]^+^: 376.1825. Found: 376.1828.

*1-(2-Fluorophenyl)-3-(3-(methylamino)-1-phenylpropyl)-1,3-dihydro-2H-benzimidazol-2-one* (**6**). Yield: 30%, yellow crystals, mp. 92 °C–93 °C, purification: silica gel 60, dichloromethane/methanol 9:1 and RP-18 silica gel methanol/water 9:1 and 7:3, ^1^H-NMR (200 MHz, CDCl_3_): δ (ppm) 2.57 (s, 3H, NHCH_3_), 3.01–3.15 (m, 4H, 2'-CH_2_, 3'-CH_2_), 5.75–5.86 (m, 1H, 1'-CH), 6.83–6.87 (m, 1H, benzim 4-CH) 6.98–7.05 (m, 3H, benzim 5-CH, benzim 6-CH, benzim 7-CH), 7.25–7.42 (m, 5H, f-phen 3-CH, f-phen 6-CH, phen 3-CH, phen 4-CH, phen 5-CH), 7.48–7.61 (m, 4 H, f-phen 4-CH, f-phen 5-CH, phen 2-CH, phen 6-CH), due to limited resolution of the instrumentation, the NHCH_3_ proton could not be detected, ^13^C-NMR (50 MHz, CDCl_3_): δ (ppm) 27.7 (2'-CH_2_), 33.2 (NHCH3), 46.9 (3'-CH_2_), 53.5 (1'-CH), 109.0 (benzim 4-CH), 110.1 (benzim 7-CH), 117.1 (d, *J* = 19.5 Hz, f-phen 3-CH), 122.2 (benzim 5-CH), 122.7 (benzim 6-CH), 125.1 (d, *J* = 3.0 Hz, f-phen 6-CH), 127.3 (phen 2-CH), 127.1 (phen 6-CH), 128.3 (phen 4-CH), 128.9 (phen 3-CH), 128.9 (phen 5-CH), 129.5 (f-phen 5-CH), 130.7 (d, *J* = 4.5 Hz, f-phen 4-CH), 136.5 (phen 1-C), 153.6 (benzim 2-CO), 157.6 (d, *J* = 250.5 Hz, f-phen 2-CF), due to limited resolution of the measuring apparatus, quaternary carbon f-phen 1-C could not be detected, MS: *m/z* (%) 375 (M^+^, 17), 318 (10), 228 (82) (M^+^-C_10_H_14_N), 199 (9), 185 (9), 147 (16), 128 (35), 117 (9), 91 (20), 58 (43), 44 (100), HRMS: *m/z* calculated for C_23_H_23_FN_3_O [M + H]^+^: 376.1825. Found: 376.1822.

### 3.3. Computational Methods

The ligand structures were built in the protonated form using Molecular Operating Environment (MOE) 2013 [42]. Homology models of human NET, SERT and DAT were obtained from the *Drosophila* dopamine transporter template (dDAT_cryst_, PDB id 4M48 [32]), by selecting the model with the most favorable Discrete Optimized Protein Energy (DOPE) of 250 generated by Modeller 9.11 [43]. The co-crystallized inhibitor nortriptyline was retained during model generation and the compounds were docked in the same site using Genetic Optimization for Ligand Docking (GOLD) 5.2 [44]. One hundred poses per ligand (*i.e.*, five hundred poses per protein target) were generated based on the GoldScore scoring function, while keeping the ligand flexible and the protein rigid.

**Figure 5:**
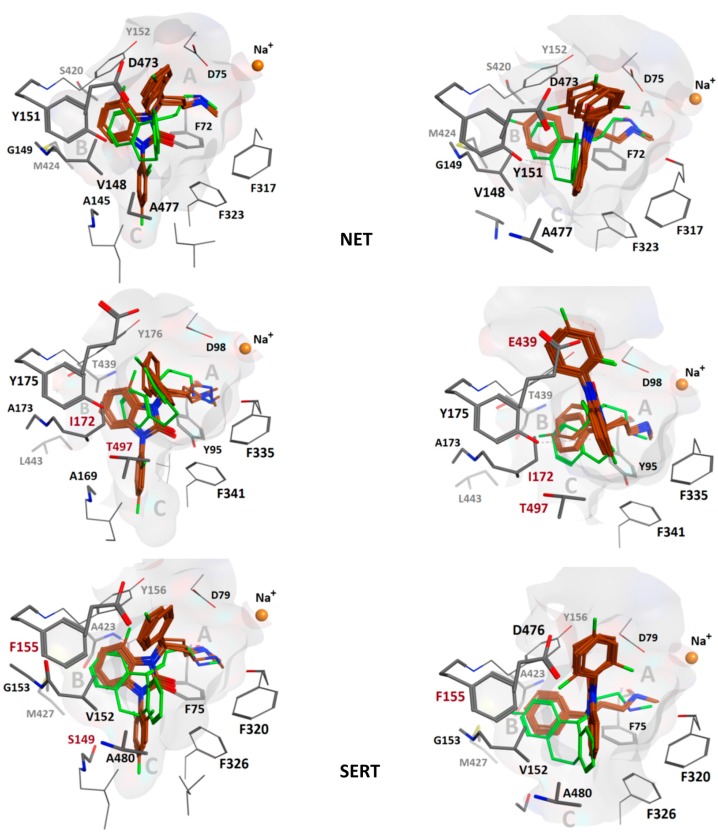
*Left column*: Overlay of compounds **1**, **2**, and **4**–**6** (maroon) in binding hypothesis 1 and comparison with the co-crystal pose of nortriptyline (**31**) (green). In NET, V148 allows more space than I172 in SERT. The angle between ligand ring 2 and 3 is *ca.* 60° in all poses. *Right column*: Overlay of compounds **1**, **2**, and **4**–**6** (maroon) in binding hypothesis 2. In NET, V148 still allows more space than in hSERT, whereas E439 might disrupt ligand ring 3. DAT lacks a more potent stacking interaction due to F155 as compared to NET[Y151] and SERT[Y175]. Binding mode 2 poses are more in agreement with the co-crystal pose. The angle between ligand ring 2 and 3 is almost 90° in all poses. The extracellular space is located above in all figures.

The common chemical scaffold, *i.e.*, the reference compound, was extracted from the resulting poses, analogous to the methods of our previous study [35]. Cluster analysis was performed based on Euclidian distance and complete linkage of the root-mean square deviation of the ligand’s heavy atoms matrix using XLStat [45]. The dendrogram was cut at eight clusters and the ones containing all five ligands were selected (Figure 5).

## 4. Conclusions

In conclusion, ten new compounds have been synthesized within the scope of this work, which aimed at the development of new, selective, high affinity references for the imaging of the NET system via PET. Four of these new compounds (**4**–**6** and **25**) will be employed in future studies. Whilst methylamines **4**–**6** (FAPPI:1-3) represent reference compounds for their later prepared radioactive analogs, additionally prepared free amine **25** (APPI:1) will serve as precursor for radiolabeling. Since docking studies indicate that fluorinated methyl amines **4**–**6** (FAPPI:1-3) bind in an analogous way to the NET as reference compound **1**, these compounds **4**–**6** are promising candidates for biological evaluation.
